# Medulloblastoma Down Under 2013: a report from the third annual meeting of the International Medulloblastoma Working Group

**DOI:** 10.1007/s00401-013-1213-7

**Published:** 2013-11-22

**Authors:** Nicholas G. Gottardo, Jordan R. Hansford, Jacqueline P. McGlade, Frank Alvaro, David M. Ashley, Simon Bailey, David L. Baker, Franck Bourdeaut, Yoon-Jae Cho, Moira Clay, Steven C. Clifford, Richard J. Cohn, Catherine H. Cole, Peter B. Dallas, Peter Downie, François Doz, David W. Ellison, Raelene Endersby, Paul G. Fisher, Timothy Hassall, John A. Heath, Hilary L. Hii, David T. W. Jones, Reimar Junckerstorff, Stewart Kellie, Marcel Kool, Rishi S. Kotecha, Peter Lichter, Stephen J. Laughton, Sharon Lee, Geoff McCowage, Paul A. Northcott, James M. Olson, Roger J. Packer, Stefan M. Pfister, Torsten Pietsch, Barry Pizer, Scott L. Pomeroy, Marc Remke, Giles W. Robinson, Stefan Rutkowski, Tobias Schoep, Anang A. Shelat, Clinton F. Stewart, Michael Sullivan, Michael D. Taylor, Brandon Wainwright, Thomas Walwyn, William A. Weiss, Dan Williamson, Amar Gajjar

**Affiliations:** 1 Department of Paediatric Oncology/Haematology, Princess Margaret Hospital for Children, Perth, WA Australia; 2 Princess Margaret Hospital for Children, Perth, Australia; 3 Telethon Institute for Child Health Research, The University of Western Australia, Perth, Australia; 4 John Hunter Children’s Hospital, Newcastle, Australia; 5 Barwon Health, Andrew Love Cancer Centre, Geelong, Australia; 6 PathWest, Perth, Australia; 7 Northern Institute for Cancer Research, Newcastle University, Newcastle, UK; 8 Curie Institute, Paris, France; 9 Stanford University School of Medicine, Stanford, CA USA; 10 Sydney Children’s Hospital Network, Sydney, Australia; 11 University of Western Australia, Perth, Australia; 12 Monash Children’s Hospital, Southern Health, Melbourne, Australia; 13 University Paris Descartes Sorbonne Paris Cite, Paris, France; 14 Royal Children’s Hospital, Brisbane, Australia; 15 Royal Children’s Hospital, Melbourne, Australia; 16 German Cancer Research Center, Heidelberg, Germany; 17 PathWest Neuropathology, Royal Perth Hospital, Perth, Australia; 18 The Children’s Hospital at Westmead, Sydney, Australia; 19 Starship Children’s Hospital, Auckland, New Zealand; 20 Fred Hutchinson Cancer Research Center, University of Washington, Seattle, WA USA; 21 Children’s National Medical Center, Washington, D.C., USA; 22 University of Bonn, Bonn, Germany; 23 Alder Hey Children’s NHS Foundation Trust, Liverpool, UK; 24 Boston Children’s Hospital, Harvard Medical School, Boston, MA USA; 25 University Medical Center Hamburg-Eppendorf, Hamburg, Germany; 26 Christchurch Hospital, Christchurch, New Zealand; 27 The Hospital for Sick Children, Toronto, Canada; 28 University of Queensland, Brisbane, Australia; 29 University of California, San Francisco, San Francisco, CA USA; 30 Department of Oncology, Mail Stop 260, St. Jude Children’s Research Hospital, 262 Danny Thomas Place, Memphis, TN 38105 USA; 31 St. Jude Children’s Research Hospital, 262 Danny Thomas Place, Memphis, TN USA

## Abstract

Medulloblastoma is curable in approximately 70 % of patients. Over the past decade, progress in improving survival using conventional therapies has stalled, resulting in reduced quality of life due to treatment-related side effects, which are a major concern in survivors. The vast amount of genomic and molecular data generated over the last 5–10 years encourages optimism that improved risk stratification and new molecular targets will improve outcomes. It is now clear that medulloblastoma is not a single-disease entity, but instead consists of at least four distinct molecular subgroups: WNT/Wingless, Sonic Hedgehog, Group 3, and Group 4. The Medulloblastoma Down Under 2013 meeting, which convened at Bunker Bay, Australia, brought together 50 leading clinicians and scientists. The 2-day agenda included focused sessions on pathology and molecular stratification, genomics and mouse models, high-throughput drug screening, and clinical trial design. The meeting established a global action plan to translate novel biologic insights and drug targeting into treatment regimens to improve outcomes. A consensus was reached in several key areas, with the most important being that a novel classification scheme for medulloblastoma based on the four molecular subgroups, as well as histopathologic features, should be presented for consideration in the upcoming fifth edition of the World Health Organization’s classification of tumours of the central nervous system. Three other notable areas of agreement were as follows: (1) to establish a central repository of annotated mouse models that are readily accessible and freely available to the international research community; (2) to institute common eligibility criteria between the Children’s Oncology Group and the International Society of Paediatric Oncology Europe and initiate joint or parallel clinical trials; (3) to share preliminary high-throughput screening data across discovery labs to hasten the development of novel therapeutics. Medulloblastoma Down Under 2013 was an effective forum for meaningful discussion, which resulted in enhancing international collaborative clinical and translational research of this rare disease. This template could be applied to other fields to devise global action plans addressing all aspects of a disease, from improved disease classification, treatment stratification, and drug targeting to superior treatment regimens to be assessed in cooperative international clinical trials.

## Introduction

Medulloblastoma is the most common malignant brain tumor of childhood. The disease affects one in five children who have a brain tumor [46]. The current World Health Organization’s (WHO) classification recognizes the classic medulloblastoma and four histological variants of medulloblastoma: anaplastic, large cell, nodular desmoplastic, and medulloblastoma with extensive nodularity (MBEN) [17]. Since the 1990s, risk stratification of medulloblastoma has been based on age, metastatic status at diagnosis, and extent of surgical resection. More recently, the presence of widespread anaplasia or large cell morphology was added to risk-stratification criteria after these cytologic phenotypes were associated with decreased progression-free survival [2, 20, 21]. In contrast, nodular desmoplastic tumors are a favorable prognostic marker in infants [42]. Children older than 3 years with localized disease at diagnosis and who have undergone complete or near-complete tumor resection (<1.5 cm^2^ residual tumor) are classified as having average-risk disease. Patients in this age group with metastatic disease at diagnosis, incomplete tumor resection, diffuse anaplasia, or large cell histology are classified as having high-risk disease. Children younger than 3 years and infants comprise a separate clinical group.

Approximately 80 % of children treated in the average-risk group are now long-term survivors. This group has a 5- to 10-year disease control rate of 75 to 85 % after treatment with the most commonly used approach, i.e., 23.4 Gy craniospinal irradiation (CSI) and a posterior fossa boost to 55.8 Gy in combination with vincristine during CSI. Radiotherapy is followed by maintenance chemotherapy using vincristine, cisplatin, and lomustine and/or cyclophosphamide [15, 32]. Survival for the high-risk group has also improved to 60–70 % [8, 11], achieved by using a higher dose of CSI (36–39.6 Gy) and intensifying chemotherapy. However, these survival figures have plateaued, with little improvement seen in the past decade [14, 31, 33, 50, 51]. Particularly troubling are the devastating long-term sequelae, including developmental, neurological, neuroendocrine, and psychosocial deficits and second tumors currently endured by survivors [16, 18, 19, 24, 25, 35, 36, 40, 47, 48].

The current explosion of molecular data has begun to elucidate the biologic makeup of medulloblastoma [26, 27, 34]. This information is expected to lead to improved disease classification, treatment stratification, and the discovery of novel drug targets. Seminal array-based transcriptional-profiling studies of large cohorts of primary medulloblastoma samples [1, 13, 28, 52] have divided medulloblastoma into at least four distinct subgroups: Wnt/Wingless (WNT), Sonic Hedgehog (SHH), Group 3, and Group 4 medulloblastoma [49]. These subgroups have distinct cytogenetic features, genetic aberrations, gene expression profiles, and divergent phenotypes including patient demographics, tumor cell histology, and outcomes [3–7, 38, 44]. More recently, single-nucleotide polymorphism (SNP) arrays and whole-genome and whole-exome sequencing have uncovered further the genetic landscape of medulloblastoma [12, 29, 37, 39, 41]. Thus, what was once regarded a single-disease entity is now recognized as being at least four distinct diseases that require individual diagnostic and therapeutic approaches. To further improve cure rates and neurodevelopmental outcomes, future prospective clinical trials will need to adopt an integrative classification system based on clinical, histopathologic, and molecular criteria. Determining these molecular subgroups at diagnosis is expected to significantly enhance risk classification and identify those patients most likely to benefit from molecularly targeted therapies, as well as therapy reduction. The rapid accumulation of molecular data poses the clinical challenge of applying this data in a timely manner in clinical trials. An important element of the next generation of medulloblastoma clinical trials will be the robust assignment of subgroup status to inform clinical and research advances.

Presented here is an account of the meeting, which was divided into four sessions: pathology and molecular stratification, genomics and mouse models, high-throughput drug screening—translating science into the clinic, and clinical trials—implementing advances into the next-generation protocols. The development and summary of a global action plan is given at the end of the report.

## Meeting report

### Session 1: Pathology and molecular stratification

David Ellison discussed the potential use of a combined molecular and histopathologic classification. Medulloblastoma is poised to be the first brain tumor to be defined primarily by molecular subgroup and histopathologic features. The new scheme must maintain clinical utility, provide a clear link with the current histopathologic classification, be based upon robust, reproducible and specific assays, and be applicable in pathology laboratories around the world. Diagnostic pathologic evaluation would be based on a combination of standard histology, immunohistochemistry (IHC), and the application of molecular testing. Concerns about the subjectivity of histologic interpretations and the morphologic heterogeneity of medulloblastoma were raised, highlighting the need for a standardized method of histologic analysis.

On the basis of previously reported clinicopathologic correlates, Dr. Ellison [4–6] suggested the following histopathology-based classification for consideration (Table 1).

**Table 1:** Suggested medulloblastoma classification based on histopathologic and molecular classification

Molecular variant	Morphologic classification
WNT subgroup	Classic, LC/A
SHH subgroup	Desmoplastic (nodular), including MBEN, classic, and LC/A
Non-WNT/non-SHH subgroup	Classic, LC/A, differentiating, melanotic, medullomyoblastoma

*LC/A* large cell/anaplastic, *MBEN* medulloblastoma with extensive nodularity, *SHH* Sonic Hedgehog, *WNT* Wnt/Wingless

Torsten Pietsch discussed the German experience with the brain tumor reference center for neuro-oncological studies and tissue bank center (Brain-Net, brain tissue bank Bonn). The center’s tasks include performing centralized reviews, providing second opinions on difficult cases, and developing new diagnostic tools and training. To minimize discordance between centers, digital “virtual” microscopy is often used to assist smaller centers, and reference pathologists meet regularly in an effort to reach a consensus about the percentage of features that constitute a certain disease classification. The process of tissue banking was discussed, and the requirements of a successful bank included a decentralized tumor repository, written and informed consent, intense coordination and cooperation with local neurosurgeons, and standardization of material preservation.

Steve Clifford discussed the use of molecular stratification of medulloblastoma moving forward. The International Society of Paediatric Oncology (SIOP) PNET 3 clinical trial was retrospectively reviewed using the GoldenGate DNA methylation array (Illumina, San Diego, CA, USA) to analyze DNA extracted from formalin-fixed, paraffin-embedded (FFPE) tumor samples. Dr. Clifford clearly showed that the four medulloblastoma subgroups can be identified using this technology [45].

Marc Remke discussed further dissection of the molecular subtypes of medulloblastoma by using methylation and expression profiling on samples from the Medulloblastoma Advanced Genomic International Consortium (MAGIC). His data showed a still-evolving molecular classification of medulloblastoma. Although he identified the four disease subgroups, Groups 3 and 4 demonstrated additional distinct subtypes. Group 3 can be further stratified into subtypes primarily based on the presence of *MYC* amplification and chromosome 8 abnormalities. Further stratification of Group 4 was less clear. Importantly, each group appears to have a different clinical outcome; thus, more work is needed to further delineate these groups and their importance to treatment.

Paul Fisher presented opportunities for epidemiologic investigation of medulloblastoma, in particular investigating germline variants across regional or national populations. By elucidating these differences, we may discover further mechanisms of disease induction and progression.

Paul Northcott outlined work investigating germline variations and their implications in the development of medulloblastoma. He raised the question of the general contribution of known cancer genes that are mutated in the medulloblastoma germline and the importance of penetrance rate. To address this, Dr. Northcott will assess the incidence of germline single-nucleotide variations and insertions/deletions of genes by investigating the frequency of causative germline copy number variants in medulloblastoma.

Brandon Wainwright presented his group’s work on dependence-receptor signaling in medulloblastoma. In particular, he discussed Neogenin1 (*NEO1*) [22], an axon-guidance molecule involved in chemoattraction during axon growth. Low levels of *NEO1* expression adversely affects prognosis in patients with SHH disease, but it is unclear how the molecule functions in this context. Dr. Wainwright stressed that *NEO1* receptors and ligands could be therapeutic targets in the future.

### Session 2: Genomics and mouse models

Michael Taylor presented a comparison of FFPE tumor samples of primary disease and relapsed tumor obtained from the same patients in a discovery cohort at the Hospital for Sick Children in Toronto. Dr. Taylor showed evidence that the molecular features of medulloblastoma do not switch between diagnosis and relapse. Furthermore, the patterns of relapse and recurrence are subgroup specific. For example, SHH medulloblastoma tends to recur locally, but Groups 3 and 4 almost always recur with metastases. There are also emerging data on genetic aberrations (e.g., cytogenetic features and copy number variations) that discern clinically significant prognostic subgroups. *GLI2* amplification, *MYCN* amplification, 14q loss, and chromothripsis indicate poor prognosis in SHH medulloblastoma, and *MYC* amplification and isochromosome 17q are negative prognostic markers in Group 3 tumors. In Group 4, patients with chromosome 11 loss or chromosome 17 gain tend to fare better and have a longer period to recurrence, but *MYCN* amplification has no prognostic effect in this subgroup. Dr. Taylor indicated that it is imperative that the genetic heterogeneity within the disease subgroups be defined. He proposed a “6-pack” of fluorescence in situ hybridization (FISH) tests (*GLI2*, *MYC*, 14q, 17p, 17q, and 11q) as a “pathology laboratory-friendly” means to delineating the disease.

Dr. Taylor also presented work from his laboratory investigating the role of membrane depolarization as a potential medulloblastoma tumor suppressor. Glutamate is expressed during normal cerebellar development and drives tachyphylaxis to SHH protein in the progenitor cells in the external granular layer. Glutamate signaling is downregulated in *Ptch* ^+/−^ mice and in human SHH medulloblastoma, compared to levels in normal cerebellum, suggesting opposing developmental roles of SHH and glutamate in normal cerebellar development and medulloblastoma.

Scott Pomeroy discussed genetic and epigenetic changes that occur during medulloblastoma tumorigenesis. He is attempting to discern how medulloblastoma subtype-specific copy number alterations affect transcription by interrogating protein–protein interaction databases. Many nonrecurrent perturbations in epigenetic machinery are found across all medulloblastoma subtypes that appear in network modules. Using these databases, Dr. Pomeroy has found high-confidence interactions that, when plotted, display SHH-subtype networks of interactions.

David Jones presented data on the methylomic landscape of medulloblastoma and discussed different techniques to determine methylation trends in the genome. The first technique discussed was the Illumina Infinium HumanMethylation450 BeadChip array (450K array) which includes >4,50,000 probes covering essentially all genes, and is sufficient to classify the subgroup of most tumor types with a better match to gene expression profiling compared with the IHC markers assessed [10]. This non-subjective assay works well with FFPE material, as confirmed by comparison with results from assays of fresh-frozen samples.

The second technique Dr. Jones discussed was whole-genome bisulfite sequencing (WGBS). This method is more expensive, requires more DNA, and is computationally more challenging than the 450K arrays. Unlike the arrays, however, WGBS covers all CpG’s; therefore, it provides a greater degree of accuracy in detecting differentially methylated regions. A large number of medulloblastoma subgroup genes show methylation-expression correlations. All WNT tumors and Group 3 tumors demonstrate hypomethylation. Additionally, these medulloblastoma subtypes almost exclusively demonstrate partially methylated domains, which consist of megabase-scale regions displaying hypomethylation overlapping with markers of heterochromatin and reduced gene expression. The fact that genes within partially methylated domains tend to be expressed at very low levels and the rate of concurrent somatic mutations is higher supports the hypothesis of compact chromatin being less accessible to DNA repair.

David Jones also presented on behalf of Marcel Kool, who is investigating SHH subgroup-outcome prediction to SMO inhibition. Within the SHH-subtype of medulloblastoma, three subgroups can be distinguished based on mutations: infants, children, and adults. Infant-SHH tumors tend to have a high rate of *PTCH* mutations and germline *SUFU* mutations, with few cases of *MYCN* and *GLI2* amplification or *TP53* mutations. Tumors from patients aged 5–16 years show frequent *TP53* mutations, which are often germline, and amplifications of *GLI2* and *MYCN*. Adult-SHH medulloblastoma tumors show greater numbers of recurrent mutations and more mutations per tumor than do pediatric tumors, and mutations in *SMO* are common in adult cases. LDE225, a SMO inhibitor currently being assessed in a Phase III trial for SHH-positive medulloblastoma (http://clinicaltrials.gov/ct2/show/NCT01708174) shows different sensitivities depending on mutation types. Primary resistance to LDE225 depends on the germline predisposition (e.g., *SUFU* mutation) or presence of *GLI2/MYCN* amplifications. These data will be crucial for drug trials to identify the appropriate candidates to receive these novel agents.

Dan Williamson discussed identifying druggable targets in a mutagenesis-modifier screen of the *Ptch* ^+/−^ medulloblastoma model (Sleeping Beauty transposon system in mice). This model demonstrated that the Sleeping Beauty mutagenesis system in *Ptch* ^+/−^ mice perturbed a transcription factor network, leading to the upregulation of *Igf2*, maintenance of medulloblastoma proliferation, and increased tumor frequency. It also highlighted the central role for Igf2 expression in medulloblastoma formation and identified Myt1L as a novel target for intervention.

Dr. Williamson’s presentation generated discussion on the role of mouse models in directing the medulloblastoma research agenda as a validation or discovery tool. Elucidating the tumorigenic event is essential to understanding the relevance of the model. A suggestion was put forward to establish a central repository of all mouse models (e.g., transgenic models, xenograft models, etc.) that would make the animals freely available to researchers worldwide and provide information about what drives each model.

### Session 3: High-throughout drug screening—translating science into the clinic

Anang Shelat discussed the HTS program at St. Jude Children’s Research Hospital. His group employs both target-based and phenotypic HTS to prioritize compounds for drug development. Dr. Shelat provided an update on an HTS campaign targeting DDX3X, a DEAD-box RNA helicase that is mutated in approximately 50 % of human WNT medulloblastomas that have been sequenced [12, 39, 41]. Although DDX3X is not a validated drug target for medulloblastoma, the lack of quality small-molecule chemical tools to probe the function of this protein justified the need for screening. Dr. Shelat also applies a phenotypic-screening strategy using the CellTitre-Glo^®^ assay (Promega, Madison, WI, USA), which is based on ATP consumption as a measure of cell proliferation and viability. The screening library was designed to cover bioactive chemical space using compounds spanning a range of drug development times: short-term (FDA-approved drugs or late-stage clinical candidates with known target or mechanism of action), intermediate-term (pharmacophore-based compounds potentially targeting novel members from well-studied protein families such as kinases or nuclear hormone receptors), and long-term (rare scaffolds potentially targeting novel targets from less-characterized protein families). The St. Jude drug-discovery pipeline consists of a number of steps, starting with building a model based on knowledge of human tumor and mouse genetics, then developing and validating an HTS-compatible assay, followed by primary screening at a single concentration of the drug, and secondary screening of dose responses. Prior to in vivo efficacy studies, positive HTS hits are first prioritized using a washout assay to assess the longevity and mechanism of action, synergy testing with each other and standard-of-care agents, and pharmacokinetic (PK) analysis including an estimation of central nervous system (CNS) penetration. Dr. Shelat discussed the variability of HTS results, which stems in part from the functional consequences of genetic variability in models, the influence of media and environmental factors on cell growth, and interoperator variability. He stressed the need to replicate HTS results in different labs to eliminate false positives early in the drug-discovery process.

Jim Olson highlighted his group’s approach to HTS, stressing that integrity is key to this process. He defined integrity as “the concept of consistency of actions, values, methods, measures, principles, expectations, and outcomes”. HTS efforts are futile unless future clinical trials are considered and the best in vivo preclinical models are identified. Many potential drug candidates kill tumor cells in vitro, but that has little clinical relevance because metabolism, oxygen levels, and cell signaling in vitro are altered from those in the tumor’s natural environment. The point was raised that nearly all targeted drugs fail to cross the blood–brain barrier and will never benefit patients with newly diagnosed disease. However, during discussions it was suggested that drugs that are effective in vitro should not be discarded too quickly because they may yet be modified to facilitate crossing the blood–brain barrier. Even if this is not achieved, these drugs may still provide important information on the mechanisms that drive tumor growth and survival.

Dr. Olson highlighted the two approaches one can take to HTS. First, HTS can be used as a tool to screen potential drugs based on their PK and pharmacologic data and identify potential therapies to translate directly into the clinic. Second, HTS can be used to increase our understanding of the biology of the tumors and then develop drugs that target the vulnerabilities identified. Dr. Olson also briefly commented on the use of knottins (small, diverse, and stable proteins) as drug scaffolds but acknowledged the many challenges associated with this approach. Knottins have a short half-life in serum, have issues with solubility, and exhibit poor folding in *Escherichia coli* and yeast. They are difficult to synthesize in the milligram quantities required, and it is expensive to produce diverse examples. In addition, knottins are not easy to use in medicinal chemistry. Finally, Dr. Olson emphasized the crucial importance of international collaboration in clinical trials to expedite drug development from patient to bench and back.

William Weiss discussed his group’s approach to *MYC*-amplified medulloblastoma. SHH and Group 3 tumors are often MYC-amplified, either nuclear or cytoplasmic. MYC is a highly stable protein that is generally thought to be undruggable, because it has no apparent surfaces for small-molecule binding. Dr. Weiss’ group is attempting to bind MYC indirectly by using PI3K-pathway blockade or aurora kinase inhibitors, which can induce a novel and distinct conformation of aurora kinase A that is unable to stabilize *MYCN*.

Jae Cho continued the MYC theme, discussing his work on the genetic and chemical perturbations of MYC using a combination of high-throughput functional genomic screens, whole-genome shRNA sequencing, and chemical genome sequencing to identify and validate targets and generate lead compounds. His group has screened nearly 2,000 compounds by using chemical biology screening and has identified the intolerance of reactive-oxygen species as a vulnerability of MYC-amplified medulloblastoma cells. Agents that produce reactive-oxygen species induced apoptosis and inhibited MYC-related gene expression signatures associated with poor outcome, while having minimal side effects on cells derived from the subventricular zone. These preliminary data need to be confirmed in in vivo systems. Other promising candidate drugs identified targeted BET bromodomains in MYC-driven tumors and DDX3X in WNT-group and SHH-group tumors.

Clinton Stewart discussed preclinical PK studies in support of medulloblastoma drug development efforts and the relevance and importance of CNS penetrance to PK. The basic tenets he outlined included the importance of getting the compound to the target, the relevant concentration and length of exposure at the target (washout experiments), and the unbound drug being the active moiety. Dr. Stewart went on to talk about model-based drug development to characterize the exposure–response relationship in preclinical models [23] and the importance of performing PK studies in tumor-bearing animals, but he also emphasized that depending upon the purpose of the study non-tumor-bearing mice could also be used. Knowing an agent’s penetrance into normal brain tissue is equally important to knowing its penetrance into brain tumor tissue. The goals of PK studies are to assess the systemic and CNS drug distribution in mouse models of pediatric brain tumors, to optimize the dosing of drug (i.e., dosage, schedule, sequence) in efficacy studies, to relate these results to in vitro efficacy studies (IC_50_ and IC_90_), and to obtain data to scale-up dosing for the candidate drugs moving into clinical trials. These goals should be organized and consistent, yet flexible. Simply due to their vast number, the efficacies of all compounds cannot be studied; thus, a robust system to prioritize and optimize compounds must be adopted. Those compounds that are demonstrated active in screens using accurate tumor models should be prioritized. When moving a compound to the clinic, it is imperative to use PK and pharmacologic studies to guide dosing in efficacy studies to identify whether brain penetration is adequate to attain the desired target exposure based upon preclinical studies. For those compounds translated into the clinic, PK studies should then be performed to confirm that the exposure-time relationship observed in preclinical models is also observed in the clinic.

### Session 4: Clinical trials—implementing advances into the next-generation protocols

Roger Packer discussed the current clinical status of medulloblastoma. In recent years, overall survival has improved in patients older than 3 years, and quality of survival has improved in infants with desmoplastic/nodular medulloblastoma. However, we still face the challenges of poor survival in patients with disseminated disease, and overall quality of survival is suboptimal for all children receiving radiotherapy plus chemotherapy. Dr. Packer discussed the importance of gross total resection, the unclear role of surgery in treating disseminated tumors, and whether the efficacy of gross total resection on long-term survival is subgroup dependent. New radiotherapy techniques were addressed, and the question of whether any subset of medulloblastoma could be adequately treated with radiotherapy alone or chemotherapy alone was raised. The role of biological chemotherapy, with respect to tumor subgroups, was also discussed.

**Fig. 1:**
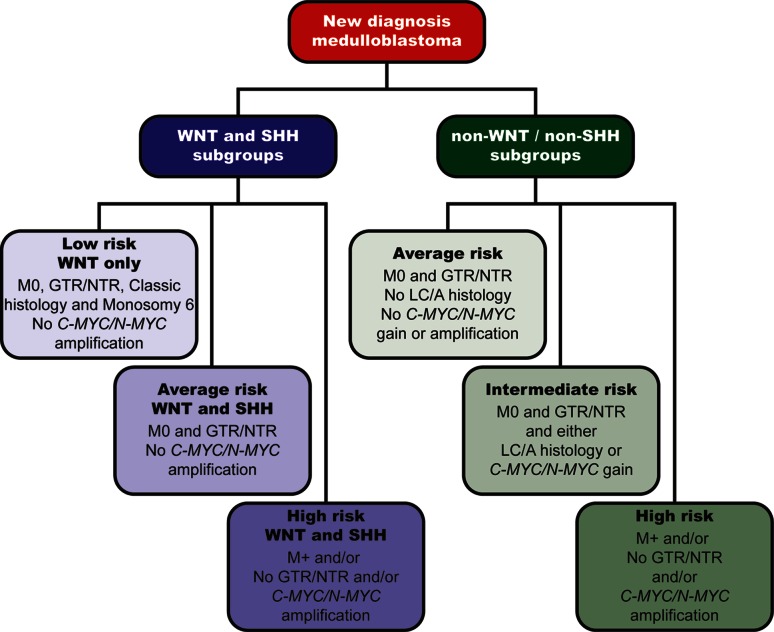
New risk criteria for medulloblastoma in recently opened St. Jude frontline medulloblastoma protocol (SJMB12)

Amar Gajjar reported on the outcome of the most recent St. Jude medulloblastoma protocol (SJMB03) in which treatment was risk stratified. This trial enrolled more than 400 patients, 330 of whom had medulloblastoma. Based on analysis of SJMB03 data, the following new risk criteria for non-infant medulloblastoma have been adopted in the recently opened successor trial called, “A Clinical and Molecular Risk-Directed Therapy for Newly Diagnosed Medulloblastoma” (SJMB12) (http://clinicaltrials.gov/show/NCT01878617) (Fig. 1).

Giles Robinson discussed exploring the new genomic and molecular findings to identify those patients most likely to suffer relapse and to use this information to develop new therapies to treat patients with relapsed medulloblastoma. He proposed a two-pronged approach to tackling this question. First, we must continue institutional and small consortium Phase I/II trials. These trials can be rapidly opened and appeal to pharmaceutical sponsors. However, problems may be encountered with small patient numbers, recruitment issues, and the long duration of such studies. Second, we must establish larger collaborative trials, which are more efficient, have larger cohorts and quicker pipelines, and address specific biologic aims. Pharmaceutical companies may be more hesitant to participate in such trials, and potential conflicts about study ownership and authorship could arise.

François Doz discussed SIOP-Europe’s new frontline average-risk medulloblastoma trial (SIOP-E PNET 5-MB), which will apply prospective biological stratification and will soon be open to enrollment. For patients older than 3–5 years, the following selection criteria will be used: posterior fossa tumor, no metastasis on CNS magnetic resonance imaging, no residual disease after surgery (or <1.5 cm^2^ largest diameter), and negative cerebrospinal fluid cytology by lumbar puncture. Patients with either LC/A medulloblastoma or *N*-*MYC/C*-*MYC* amplification are not eligible for this study. Stratification into low- or standard-risk groups is based on the presence or absence of WNT features (defined by β-catenin nuclear immunopositivity by IHC and/or β-catenin mutation analysis) and low-risk clinical features. Patients with low-risk medulloblastoma (WNT-medulloblastoma) will receive 23.4 Gy CSI with a tumor bed boost to 55.4 Gy, followed by reduced maintenance chemotherapy. Those with standard-risk disease will be randomized to receive either radiotherapy alone or radiotherapy in combination with carboplatin similar to the treatment regimen of the Children’s Oncology Group (COG) pilot study, with both treatment arms followed by maintenance chemotherapy.

Nicholas Gottardo outlined the COG-proposed front-line trial, a molecularly based stratification-feasibility study for children with newly diagnosed, average-risk medulloblastoma. Three guiding principles were established before the study was designed: (1) work toward synchronizing all patients with medulloblastoma under one study for future biology-based stratification; (2) improve the survival rate with minimal additional adverse effects and reduced long-term sequelae; (3) replace toxic agents with treatments that are less toxic yet still effective. This trial will provide the foundation for future Phase III studies of medulloblastoma by introducing molecular analysis. The study will assess the feasibility of real-time molecular stratification and central pathology review. On the basis of currently applied clinical-risk criteria, histopathologic features, and molecular assessments, patients will be classified into two molecular subgroups: WNT-driven average-risk (defined by β-catenin nuclear immunopositivity by IHC and monosomy 6 by FISH) and non-WNT-driven average-risk medulloblastoma. As in the SIOP-E PNET 5-MB study, patients with either LC/A medulloblastoma and/or *N*-*MYC/C*-*MYC* amplification will not be eligible for this study. The study will incorporate a reduced-therapy strategy for patients with WNT-driven medulloblastoma. Upon completion of the feasibility components of the study, enrollment will continue for patients with average-risk, WNT-driven medulloblastoma.

Stefan Rutkowski discussed the treatment, concepts, subgroups, and challenges of medulloblastoma in young children and infants. CSI-free survival and neurocognition have become important outcome measures especially in young children with desmoplastic medulloblastoma or MBEN, who have a better prognosis than young children with nondesmoplastic tumors [43]. Performing randomized, controlled trials can help to further improve treatment results, but the conduct of such trials in relatively rare subgroups is challenging. Based on the results of a national phase II trial [42], the PNET working group of SIOP-E plans to initiate a randomized European trial for young children with desmoplastic medulloblastoma/MBEN with systemic chemotherapy with or without intraventricular methotrexate. Based on the results of a French pilot study, a randomized trial for treating young children with nondesmoplastic medulloblastoma with induction chemotherapy, high-dose chemotherapy, and risk-adapted radiotherapy is also under development. Dr. Rutkowski encouraged international collaboration for studying this very small group of patients. He also discussed the need for better translation of the underlying biology into clinical trials and the need for inclusion criteria to be consistent across study groups.

Stefan Pfister presented data showing that patients with TP53-SHH medulloblastoma have better overall survival after receiving standard therapy than after receiving high-intensity treatment, regardless of the presence of metastatic disease. Therefore, this small but distinct subgroup of patients requires a different treatment approach, and given the very small number of patients affected by this disease, an international trial is warranted.

The last speaker of the meeting was Barry Pizer, who discussed approaches to relapsed disease. He highlighted the plight of patients with relapsed disease after previous CSI, noting that the majority have disseminated disease. Despite receiving very intensive salvage regimens, including megatherapy with autologous stem cell rescue, patients have very little chance of cure [9]. Dr. Pizer presented data from four consecutive COG studies from 1989 that included a total of 620 patients. Of the 444 patients in the average-risk group, 87 (19 %) experienced disease relapse, as did 80 (45 %) of the 176 in the high-risk group. Dr. Pizer noted that the high-risk group experienced relapse earlier. Five years after relapse, only 12 (7.2 %) patients were alive: 8 had suffered a local relapse, and 4 experienced a component of distant relapse. Similarly, of 340 children treated on the HIT-SIOP PNET 4 trial [15], 72 (20 %) suffered relapse, and only 8 (11 %) were alive at 5-year follow-up (5, alive with disease; 3, no evidence of disease).

## Development of a medulloblastoma global action plan

**Fig. 2:**
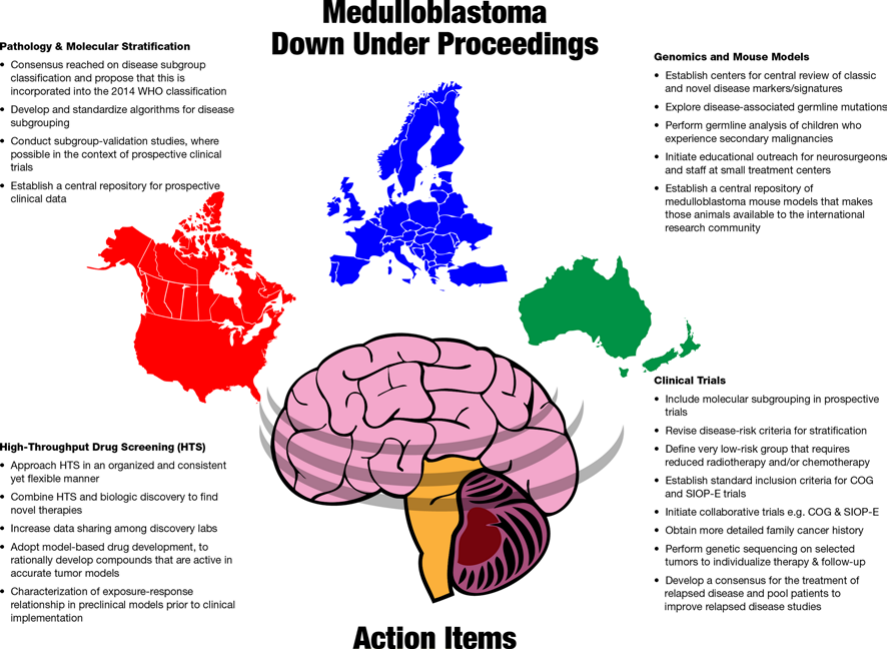
Medulloblastoma Down Under proceedings action plan

In this section, discussions that took place at the end of each session and led to the formation of a medulloblastoma global action plan (Fig. 2) are summarized. A consensus was reached in several key areas, with the most important being that a novel classification scheme for medulloblastoma based on the four molecular subgroups, as well as histopathologic features, should be presented for consideration in the upcoming fifth edition of the World Health Organization’s classification of tumours of the central nervous system. Three other notable areas of agreement were as follows: (1) to establish a central repository of annotated mouse models that are readily accessible and freely available to the international research community; (2) to institute common eligibility criteria between COG and the SIOP-E and initiate joint or parallel clinical trials; (3) to share preliminary HTS data across discovery labs to hasten the development of novel therapeutics.

### Summary of the pathology and molecular stratification session

Dr. Pfister discussed data from his laboratory, which has identified a 20 % discordance rate between standard IHC methods and newer technologies (e.g., Nanostring and 450K array profiling). Given this and the fact that IHC markers cannot reliably distinguish between the four subgroups, the International Medulloblastoma Working Group is considering whether using either of the new technologies should be standardized to aid in diagnosis and prognostication. To address this, the COG-proposed front-line trial will prospectively evaluate novel diagnostic techniques [10, 30, 45], in parallel with established (IHC and FISH) methodologies. In addition, the need to mandate subgroup classification at diagnosis and the value of this classification for patients with relapsed disease to permit their enrollment on subgroup-specific targeted therapies was discussed. A motion was made that the published consensus molecular classification of medulloblastoma [49] should be adopted. Clear agreement was reached for a 4-group classification scheme to be proposed for inclusion in the next edition of the WHO guidelines. Those groups will include WNT, SHH, Group 3, and Group 4 based on a combination of genomic, IHC, and traditional histologic evaluations. A fifth group, designated “not otherwise specified” or similar, could be included for rare tumors with a focal melanotic or rhabdomyoblastic phenotype, because these rare variants appear to fall outside the other four molecular subgroups (Robinson and Ellison, unpublished), and for those tumors that are difficult to subclassify because of assay or tissue-sampling limitations. The suggested medulloblastoma 4-group classification algorithm is shown in Table 2.

**Table 2:** Suggested algorithm for molecular classification of medulloblastoma

Medulloblastoma subgroup	Classification algorithm
WNT	Two of the following four must be met: 1. β-catenin mutation or β-catenin^+^ IHC and/or 2. Monosomy 6 and/or 3. Methylation-profiling pattern consistent with WNT or 4. Gene expression pattern consistent with WNT
SHH	Two of the following four must be met: 1. GAB1 antibody and/or 2. SHH signaling-specific mutation and/or 3. Methylation-profiling pattern consistent with SHH or 4. Gene expression pattern consistent with SHH
Group 3	One of the following must be met: 1. Methylation-profiling pattern or 2. Gene expression pattern consistent with Group 3
Group 4	One of the following must be met: 1. Methylation-profiling pattern or 2. Gene expression pattern consistent with Group 4

*IHC* immunohistochemistry, *SHH* Sonic Hedgehog, *WNT* Wnt/Wingless

The group agreed that the use of the proposed “6-pack” of FISH markers could also be considered for inclusion in the new WHO classification system, following validation in additional clinically defined cohorts. The importance of the clustering algorithms chosen to identify the biologic signals driving the groupings was highlighted, and attendees agreed that an independent validation cohort is needed to achieve consensus.

There was a great deal of discussion on whether blood and/or saliva samples should be included in baseline testing of the genomes of patients enrolled in future clinical trials to advance this evaluation. Analysis of patients on the basis of racial background was also discussed. The group agreed that, aside from the issues of self-reporting, there simply are not enough patients to achieve statistical power, and to date, no clear evidence has suggested that ethnicity affects outcome.

The SIOP-E PNET 5-MB trial will mandate both FFPE and fresh-frozen samples to support molecular diagnostics for risk stratification and future research studies. There was much debate about whether fresh-frozen tissue should be a requisite for trial enrollment. The pros and cons are detailed in Table 3. Many believed that patients should not be excluded based on whether their care facilities have the capacity to obtain, store, and ship fresh-frozen tissue. Because FFPE tumor samples provide reliable results on 450K array testing, mandating submission of fresh-frozen tissue raised an ethical conflict, given the implications that knowledge of subgroup has the potential to more accurately guide treatment (e.g., reduced intensity of CSI in patients with WNT medulloblastoma). Application of this exclusion criterion may also result in loss of statistical power, particularly in the case of the rarer medulloblastoma subtypes. Most attendees felt that with proper education both sample types could be attained and would provide optimal material for biological studies. Simon Bailey highlighted a pilot project in the UK that is looking at this question. He reported that the response from oncologists, neurosurgeons, and neuropathologists has been very good.

**Table 3:** Pros and cons of mandating fresh-frozen tissue for enrollment on a therapeutic medulloblastoma clinical trial

Pros	Cons
Support molecular diagnostics given risk of failure of molecular risk evaluation using FFPE specimens or development of a novel molecular test at a later point	Lower recruitment with consequent loss of statistical power
Optimal material for ongoing and future biologic discovery studies	Potential ethical conflict
Provide validated data for new prognostic factors and therapeutic targets	Variable capacity across institutions to obtain, store, and ship fresh-frozen tissue

*FFPE* formalin-fixed, paraffin-embedded

In the end, the group agreed that submitting fresh-frozen samples and FFPE tissue samples is preferable and should be highly encouraged. Given the non-negligible risk of failure of molecular risk evaluation using FFPE specimens and the importance for ongoing biologic discovery during prospective studies to provide validated data for new prognostic factors and therapeutic targets, fresh-frozen tissue will continue to be mandated for enrollment on the SIOP-E PNET 5-MB study. In addition, the proposed COG front-line average-risk medulloblastoma clinical trial will mandate fresh-frozen tumor tissue.

### Summary of the genomics and mouse models session

The group agreed that centers for central review of classic and novel disease markers/signatures should be established. To this end, the SIOP-E PNET 5-MB study and the proposed COG front-line average-risk medulloblastoma clinical trial will both use central reference laboratories based in Europe and the USA, respectively. To support ongoing biological studies as part of prospective clinical trials, educational outreach for neurosurgeons and staff at smaller centers should be initiated.

The group also agreed that disease-associated germline mutations should be explored and germline analysis should be performed for those children who experience secondary malignancies. Preclinical models should continue to be developed for assessment of targeted therapy, and a central repository or data system is needed for modeling medulloblastoma. Jim Olson has already begun preparing a web-based system of his laboratory’s available mouse models for sharing and has volunteered to expand this effort to the global medulloblastoma community. It was proposed that the existing complete datasets should be combined, and information should be freely available as a means of increasing the breadth of knowledge about childhood medulloblastoma, thereby facilitating our common goal of understanding this disease.

### Summary of the high-throughout drug screening: translating science into the clinic session

Discussion centered on prioritizing compounds, and although microdialysis disrupts the blood–brain barrier, it was advocated as a robust means of assessing blood–brain barrier penetrance. Setting up a microdialysis system is not a trivial endeavor; therefore, collaborations with centers that have core facilities to support such studies were encouraged. The group agreed that selecting important targets will be essential to success in this heterogeneous patient population. Not every medulloblastoma is similar and informed; thus, model-based drug development in well-understood models must be used to test the appropriate compounds. Prior to clinical implementation, the exposure–response relationship should be characterized in preclinical models.

The concept of open-source chemistry was suggested to share promising compounds at an earlier stage in development. This approach would facilitate more thorough assessments by multiple investigators using several different models and increase our knowledge of the mechanism of action and efficacy of new compounds. We must be expeditious in drug development, which will ultimately lead to more robust preclinical testing of compounds and better concordance between laboratory groups. Concern was voiced that as medulloblastoma is further subdivided, the rarity of some subgroups may limit our ability to adequately power clinical trials to assay responses in small subgroups. Thus, in addition to developing subgroup-specific therapies, we should also develop broad-ranging agents.

### Summary of the clinical trials: implementing advances into the next-generation protocols session

As a result of new information that came to light during the previous session and given the increasing awareness of the contribution of predisposing germline mutations to medulloblastoma, the group agreed that going forward, all medulloblastoma clinical trial protocols should include obtaining detailed family cancer histories from each participant. A number of general issues that need to be considered when contemplating altering clinical treatments were also discussed. These include proof of benefit, safety of the novel treatment compared to that of conventional treatments, potential neurocognitive effects, damage to the neuronal niche, and whether the treatment would be appropriate for primary versus secondary tumors.

There was little question that treatment must be assessed in molecularly defined subsets of patients, and given that study cohorts are most likely to include multiple subgroups, this will require large patient numbers. Therefore, international collaborations, if they can be undertaken efficiently, will greatly facilitate this approach. The group agreed that a common set of inclusion and response criteria for COG and SIOP-E trials should be established, and joint or parallel clinical trials should be initiated. WNT-medulloblastoma represents an ideal subtype of disease to investigate jointly. Researchers studying non-Hodgkin lymphoma, osteosarcoma, and hepatoblastoma have set a precedent for global/transatlantic studies; however, those studies were challenging due to clinical trials regulations.

Discussions then focused on future studies addressing the natural history of relapse and the dangers of selection bias when analyzing data from individual reports. Whether a curative approach using conventional therapy is reasonable in some patients was debated. If so, the determinants that justify this approach (e.g., biology or pattern of relapse) must be defined. For those patients with relapsed disease who have not undergone CSI, the treatment strategy is fairly clear. In the case of disseminated disease at relapse after CSI, most felt that a second course of radiotherapy or surgery is not recommended, but no consensus was reached. For those patients who suffer a localized relapse (posterior fossa or metastatic), most attendees agreed that a second surgical resection is appropriate if followed by adjuvant therapy. The key question is—what adjuvant therapy should be used?

Enrolling all patients who experience relapse into early-phase clinical trials was discussed; however, at present this is not in practice. Given the various molecular subtypes of medulloblastoma, it will be important to ensure that patients with relapsed disease receive subgroup-appropriate therapies (e.g., SMO inhibitors or future DDX3X inhibitors). The MEMMAT (Medulloblastoma European Multiagent Metronomic Antiangiogenic Trial) (http://clinicaltrials.gov/show/NCT01356290) was briefly discussed, and all agreed that we must develop a consensus on the treatment of relapsed disease and standardize quality-control procedures, eligibility, and treatment-response criteria between the COG and SIOP-E trials.

The group also discussed collecting tumor samples at autopsy. Most centers do not collect such samples. Tumor samples obtained at autopsy are not as fresh as those obtained during surgical resection, and yeast contamination may be a problem; however, the tissue samples are still valuable, especially if collected within 12 h of death. Samples collected during this period provide adequate amounts of good-quality DNA and RNA. Sample collection as much as 24 h after death is acceptable for xenograft models of aggressive tumors, despite low cell viability. Directly establishing cell lines from tumor samples obtained 24 h after death can, however, be very challenging. Therefore, generating cell lines from xenograft model tumors may be a better approach.

Finally, the value of Phase 0 trials for localized or disseminated medulloblastoma was discussed. Mandating treatment before brain biopsy and continuing treatment thereafter was also suggested.

## Conclusion and future directions

Medulloblastoma Down Under 2013 brought together leading researchers and clinicians to develop a global action plan to defeat this disease. The combination of thought-provoking presentations and intensely focused working groups resulted in the development of our Medulloblastoma Global Action Plan, which outlines the key areas in which a consensus was reached for what needs to be done to move the field forward. We need to classify medulloblastoma into the four molecular subgroups; WNT, SHH, Group 3, and Group 4. The techniques adopted to do this must be reliable and globally applicable. Currently, molecular subgroups are clinically informative for the WNT and SHH subgroups only, because rational individual therapies for Group 3 and Group 4 do not exist. Therefore, data on these latter two subgroups still need to be collected as a basis for future developments. It is also paramount that molecular discovery of additional medulloblastoma subgroups be linked to clinical parameters so that such subgroups are clinically relevant.

Despite intense debate, a number of important issues remained unresolved at the conclusion of the meeting. These include mandating fresh-frozen tissue for enrollment in clinical trials, the optimal technology to analyze specimens (Illumina 450K array vs. Nanostring diagnostics vs. WGBS vs. GoldenGate DNA methylation assay) and when to introduce novel targeted therapies (at diagnosis or upon relapse of disease). These are all important points that must be considered and will be addressed again at the 2014 Meeting of the Medulloblastoma Working Group and beyond, until a consensus is reached.

In conclusion, the progress made at this meeting highlights the value of international working groups as a novel vehicle for collaborative research. This template could be applied to other fields to devise global action plans addressing all aspects of the disease of interest, from improved disease classification, stratification, and drug targeting to superior treatment regimens to be assessed in collaborative international clinical trials. As new medulloblastoma subtypes are identified, the patient population of each subgroup will decrease, making it more difficult to recruit sufficient numbers of patients to determine optimal therapy. The group agreed that the medulloblastoma research community must work together to overcome regulatory, geographical, and competitive barriers and initiate joint clinical trials. Unless we find a way to overcome these hurdles, we will struggle to expeditiously identify and introduce optimal therapies into clinical practice.
